# Frequency of Anemia in Patients With Heart Failure With Preserved Ejection Fraction (HFpEF)

**DOI:** 10.7759/cureus.103066

**Published:** 2026-02-05

**Authors:** Noman Ahmed, Kaleemullah Shaikh, Akshy Kumar, Ekta Bai, Simran Kour, Faryal Fatima

**Affiliations:** 1 Department of Cardiology, Liaquat National Hospital, Karachi, PAK; 2 Department of Physiotherapy, Sindh Institute of Cardiovascular Diseases, Sukkur, PAK; 3 Department of Medicine, Shaheed Mohtarma Benazir Bhutto Medical University, Larkana, PAK; 4 Department of Medicine, Ghulam Muhammad Mahar Medical College, Sukkur, PAK

**Keywords:** anemia, echocardiography, ejection fraction, heart failure, hematology

## Abstract

Background: Cardiovascular disease remains a major health burden in Pakistan, with heart failure (HF) contributing substantially to morbidity. Anemia is a common yet underrecognized comorbidity in patients with heart failure with preserved ejection fraction (HFpEF).

Objective: The primary objective of this study is to determine the frequency of anemia in patients with HFpEF* *and the secondary objectives are to (i) assess the association between anemia and clinical comorbidities, including hypertension, diabetes mellitus, and chronic kidney disease, and (ii) evaluate the relationship between anemia and markers of HF severity, including NT-proBNP, E/e′ ratio, and left ventricular ejection fraction.

Methodology: From June to December 2024, a prospective cross-sectional study was conducted at Liaquat National Hospital, involving patients diagnosed with HFpEF. Participants were recruited through purposive non-probability sampling from the tertiary heart failure inpatient department. Data of various clinical and demographic variables were systematically recorded using a pre-designed proforma.

Results: Among 102 HFpEF patients, the mean age was 67.38 ± 8.99 years. Female patients comprised 58.8% (n = 60), while male patients accounted for 41.2% (n = 42). Anemia was detected in 76% (n = 72) of the population. Diabetes mellitus was present in 57.8% (n = 59) and hypertension in 84.3% (n = 86) of participants. Within the anemic subgroup (n = 78), 75% (n = 59) were elderly, and female patients represented the majority, indicating a gender- and age-linked predisposition.

Conclusion*:* Anemia is highly prevalent among HFpEF patients and is commonly associated with advanced age and comorbid conditions, highlighting the need for routine screening and early management to improve clinical outcomes.

## Introduction

Cardiovascular disease (CVD) remains a major global health burden [1,2] and poses a significant public health challenge in Pakistan. CVD, especially heart failure (HF), is increasing in Pakistan, reflecting worldwide patterns [3]. HF is recognized as a worldwide epidemic, with rising prevalence, impacting around 5.7 million people in the United States and being the primary cause of hospitalization in older adults [4,5]. In patients with HF, heart failure with preserved ejection fraction (HFpEF) is gaining more recognition [6]. Anemia frequently coexists in this group, particularly in older individuals, with its prevalence rising as age increases [7]. The development of anemia in HF is multifactorial and may result from chronic inflammation, impaired renal perfusion leading to reduced erythropoietin production, hemodilution due to fluid retention, nutritional deficiencies, and iron dysregulation. These interrelated mechanisms form the basis of the cardio-renal-anemia syndrome, in which cardiac dysfunction, renal impairment, and anemia exacerbate one another, contributing to disease progression [8,9]. Clinically, anemia often presents subtly in patients with chronic illness, resulting in underdiagnosis. Evidence indicates that anemia in HF is associated with higher mortality, increased hospitalizations, reduced functional capacity, and poorer quality of life [10-13]. The prevalence of anemia in HF subjects presenting with a preserved ejection fraction is described in a wide range to be 19-68% [14]. Anemia was found in around 37% of HF patients, with 57% of them being male. These results align with earlier national and global research [15]. In countries such as Pakistan, where anemia rates are elevated, it is crucial to incorporate anemia screening and treatment into standard HF care [16]. Research by Iftekhar et al. showed that anemia in HF serves as a crucial prognostic indicator, linked to higher mortality and increased rehospitalization rates [8].

Anemia has been associated with indicators of diastolic dysfunction, including echocardiographic measures like the E/A and E/e' ratios, along with increased right atrial pressure and pulmonary artery systolic pressure. These connections highlight the role of anemia in negative cardiovascular results [17]. Similarly, Pinter et al. observed that anemia was a significant contributing factor to the high prevalence of comorbidities in HF patients with mildly reduced ejection fraction. Anemia, which can serve as an independent predictor of mortality, was identified as the most impactful comorbidity. Moreover, regardless of the type of HF, anemia negatively affected clinical outcomes in patients with an ejection fraction greater than 40%.

These findings highlight the importance of early detection and management of anemia in individuals with HF [18]. In this context, we aimed to investigate the frequency of anemia in patients with HFpEF. The objectives of the study are to (i) determine the frequency of anemia among patients with HFpEF presenting to a tertiary care center, (ii) assess the association between anemia and clinical comorbidities, including hypertension, diabetes mellitus, and chronic kidney disease, and (iii) evaluate the relationship between anemia and markers of HF severity, including NT-proBNP, E/e′ ratio, and left ventricular ejection fraction (LVEF).

## Materials and methods

This prospective cross-sectional study was conducted at the Department of Cardiology, Liaquat National Hospital, Karachi, from June to December 2024. The minimum required sample size was calculated using the World Health Organization sample size calculator, assuming a prevalence of anemia of 53%, a 95% confidence level, and a 10% margin of error, yielding a required sample of 96 patients. To improve study power and account for potential exclusions, a total of 102 eligible patients were ultimately enrolled. Participants were recruited using purposive non-probability consecutive sampling from the tertiary heart failure inpatient department. Patients aged 18 years or older with a confirmed diagnosis of heart failure with preserved ejection fraction (LVEF ≥ 50% on echocardiography), fulfilling clinical and echocardiographic criteria for HFpEF, and willing to provide informed consent were included in the study. Patients were excluded if they declined participation, had incomplete clinical or laboratory records, or had identifiable alternative causes of anemia such as acute bleeding, hematological disorders, or chronic inflammatory or malignant conditions requiring separate diagnostic evaluation.

Data collection 

After obtaining written informed consent, demographic and clinical information were recorded using a structured pre-designed proforma (Appendix). Data collected included age, gender, height, weight, body mass index, systolic and diastolic blood pressure, smoking status, and comorbidities such as hypertension, diabetes mellitus, chronic kidney disease, and family history of ischemic heart disease. All patients underwent detailed clinical examination and echocardiography to document LVEF fraction and diastolic function parameters, including the E/e′ ratio. Cardiac biomarkers including Troponin I and NT-proBNP were measured using standard hospital laboratory techniques. Venous blood samples were collected to measure hemoglobin levels and renal function parameters. Anemia was defined according to World Health Organization criteria as hemoglobin <13 g/dL in men and <12 g/dL in women. Serum creatinine was measured, and estimated glomerular filtration rate (eGFR) was calculated using the CKD-EPI equation to assess renal function. All data were checked for completeness and accuracy prior to entry into the database. Potential confounding factors were minimized through stratification based on age, gender, BMI, and comorbid conditions. The primary outcome was the frequency of anemia among HFpEF patients. Secondary outcomes included the association of anemia with demographic characteristics, clinical comorbidities, cardiac biomarkers, echocardiographic measures of diastolic dysfunction, and renal function parameters.

Statistical analysis

Data were analyzed using IBM SPSS Statistics for Windows, Version 27 (Released 2019; IBM Corp., Armonk, New York, United States). Quantitative variables were expressed as mean ± standard deviation, while qualitative variables were presented as frequencies and percentages. An independent samples t-test was used to compare continuous variables between anemic and non-anemic groups. The chi-square test was applied for categorical variables, and Fisher’s exact test was used when expected cell counts were ≤5. One-way ANOVA was performed for comparisons across multiple diastolic dysfunction categories. A p-value ≤ 0.05 was considered statistically significant.

## Results

A total of 102 patients diagnosed with HFpEF were enrolled in the study. The mean age of the participants was 67.38 ± 8.99 years, with female patients accounting for 60 (58.8%) of the cohort. Among the study population, 86 (84.3%) had a history of hypertension, 59 (57.8%) were diagnosed with diabetes mellitus, and 72 (70.6%) were found to have anemia. Within the anemic subgroup, 75% were older and predominantly female. This subgroup also exhibited higher systolic blood pressure and elevated levels of cardiac biomarkers, specifically mean Troponin I and NT-proBNP (Table 1).

**Table 1:** Demographic profile and clinical features of the study participants

Variable	Value
Age (years)	67.38 ± 8.99
Male gender	42 (41.2%)
Female gender	60 (58.8%)
Age ≤ 60 years	21 (20.6%)
Age 61–80 years	75 (73.5%)
Age > 80 years	6 (5.9%)
Systolic BP (mmHg)	124.98 ± 17.71
Diastolic BP (mmHg)	72.75 ± 10.25
Left Ventricular Ejection Fraction (%)	53.99 ± 2.49
NT-proBNP (pg/mL)	6306.86 ± 8640.59
E/e′ Ratio	15.38 ± 3.70
Hypertension	86 (84.3%)
Diabetes Mellitus	59 (57.8%)
Chronic Kidney Disease	8 (7.8%)
Current Smokers	12 (11.8%)
Hemoglobin (g/dL)	11.05 ± 2.27

Anemia was more common in female patients (55.6%) compared to male patients (44.4%); this difference was not significant (χ² = 1.08, p = 0.299). Anemic patients were slightly older (67.97 ± 9.17 years) than non-anemic patients (65.96 ± 8.53 years), but the difference was not meaningful (t = 1.02, p = 0.307). Clinical parameters, including systolic and diastolic blood pressures, troponin I levels, LVEF, NT-proBNP, and E/e′ ratio, were similar between the two groups, with all p-values >0.05. Age group distribution also revealed no significant association with anemia status (χ² = 0.29, p = 0.864). While diastolic dysfunction severity appeared numerically worse in anemic patients (>14 in 73.6% vs. 66.7%), it did not reach statistical significance (χ² = 3.44, p = 0.062). Comorbidities, including hypertension, diabetes mellitus, chronic kidney disease, family history of ischemic heart disease, and smoking status, showed no significant associations with anemia (all p >0.05) (Table 2).

**Table 2:** Comparison of Demographic, Clinical, and Laboratory Variables Between Anemic and Non-anemic Patients With Corresponding Test Statistics (n = 102) An independent samples t-test was used for continuous variables (means ± SD) including age, SBP, DBP, Troponin I, LVEF, NT-proBNP, and E/e′ ratio. The chi-square test (χ²) was applied for categorical variables including gender, age groups, Troponin I groups, comorbidities, and smoking status. Fisher’s exact test was applied when expected cell counts were ≤5 (e.g., family history of IHD). A p-value ≤ 0.05 was considered statistically significant.

Variable	Anemia Yes	Anemia No	Test Statistic	p-value
Gender	32 (44.4%)	10 (33.3%)	χ² = 1.08	0.299
Female	40 (55.6%)	20 (66.7%)	—	—
Age (years)	67.97 ± 9.17	65.96 ± 8.53	t = 1.02	0.307
Age Groups				
≤60 years	14 (19.4%)	7 (23.3%)	χ² = 0.29	0.864
61–80 years	54 (75.0%)	21 (70.0%)	—	—
>80 years	4 (5.6%)	2 (6.7%)	—	—
Systolic BP (mmHg)	126.50 ± 15.93	121.33 ± 21.27	t = 1.35	0.181
Diastolic BP (mmHg)	72.79 ± 9.06	72.66 ± 12.84	t = 0.06	0.956
Troponin I (ng/mL)	0.45 ± 1.27	1.37 ± 4.59	t = 1.07	0.286
Troponin I Groups				
<0.1	49 (68.1%)	19 (63.3%)	χ² = 2.05	0.358
0.1–24.99	23 (31.9%)	10 (33.3%)	—	—
>25	0 (0%)	1 (3.3%)	—	—
Left Ventricular Ejection Fraction (%)	54.01 ± 2.14	53.93 ± 3.21	t = 0.15	0.883
NT-proBNP (pg/mL)	6599.90 ± 8974.84	5603.56 ± 7879.97	t = 0.53	0.598
E/e′ Ratio	15.27 ± 3.77	15.63 ± 3.60	t = 0.44	0.661
Diastolic Dysfunction Grade				
≤8	7 (9.7%)	0 (0%)	χ² = 3.44	0.062
9–14	12 (16.7%)	10 (33.3%)	—	—
>14	53 (73.6%)	20 (66.7%)	—	—
Hypertension	61 (84.7%)	25 (83.3%)	χ² = 0.0007	1
Diabetes Mellitus	38 (52.8%)	21 (70.0%)	χ² = 2.56	0.109
Chronic Kidney Disease	5 (6.9%)	3 (10.0%)	χ² = 0.16	0.69
Family History of IHD	1 (1.4%)	0 (0%)	Fisher’s Exact	1
Smoking Status				
Current smoker	7 (9.7%)	5 (16.7%)	χ² = 1.03	0.598
Ex-smoker	4 (5.6%)	1 (3.3%)	—	—
Non-smoker	61 (84.7%)	24 (80.0%)	—	—

Gender demonstrated a significant association with dysfunction grade, with all patients in the ≤8 category being male, while female patients predominated in the 9-14 and >14 groups (p = 0.004). Age and blood pressure values did not differ significantly across categories. NT-proBNP levels increased progressively with worsening dysfunction, rising from 243.14 ± 2.79 pg/mL in the ≤8 group to 7685.16 ± 9571.13 pg/mL in the >14 group (p = 0.024). Similarly, E/e′ ratios showed a marked stepwise rise across categories, confirming significantly higher filling pressures with greater dysfunction (p < 0.001). Diabetes mellitus was significantly more prevalent in moderate and severe dysfunction groups (p = 0.004), whereas hypertension, smoking status, chronic kidney disease, and family history of IHD did not show significant associations (Table 3).

**Table 3:** Distribution of Demographic, Clinical, and Laboratory Variables Across HFpEF Dysfunction Severity Categories (n = 102) χ²: Chi-square test for categorical variables; F: one-way ANOVA test statistic for continuous variables Fisher’s exact test was used when expected cell counts ≤5; p ≤ 0.05 considered statistically significant; * indicates a statistically significant result

Variable	≤8 n (%) / Mean ± SD	9–14 n (%) / Mean ± SD	>14 n (%) / Mean ± SD	Test Statistic	p-value
Gender				χ² = 11.02	0.004*
Male	7 (100%)	8 (36.4%)	27 (37.0%)		
Female	0 (0%)	14 (63.6%)	46 (63.0%)		
Age (years)	65.57 ± 3.30	67.22 ± 5.69	67.60 ± 10.13	F = 0.17	0.849
Age Groups				χ² = 2.82	0.246
≤60	0 (0%)	3 (13.6%)	18 (24.7%)		
61–80	7 (100%)	19 (86.4%)	49 (67.1%)		
>80	0 (0%)	0 (0%)	6 (8.2%)		
Systolic BP (mmHg)	125.42 ± 15.30	119.59 ± 13.62	126.56 ± 18.85	F = 1.32	0.272
Diastolic BP (mmHg)	70.00 ± 1.63	72.13 ± 10.61	73.20 ± 10.64	F = 0.36	0.7
Troponin I (ng/mL)	0.09 ± 0.00	0.51 ± 1.06	0.84 ± 3.15	—	—
Troponin I Groups				χ² = 2.65	0.268
<0.1	7 (100%)	13 (59.1%)	48 (65.8%)		
0.1–24.99	0 (0%)	9 (40.9%)	24 (32.9%)		
>25	0 (0%)	0 (0%)	1 (1.4%)		
LVEF (%)	54.00 ± 1.91	54.90 ± 2.95	53.71 ± 2.34	F = 2.00	0.142
NT-proBNP (pg/mL)	243.14 ± 2.79	3662.77 ± 4337.54	7685.16 ± 9571.13	F = 4.02	0.024*
E/e′ Ratio	6.85 ± 0.89	11.86 ± 1.61	17.26 ± 2.04	F = 118.7	<0.001*
Hypertension				χ² = 2.29	0.317
Yes	7 (100%)	20 (90.9%)	59 (80.8%)		
No	0 (0%)	2 (9.1%)	14 (19.2%)		
Diabetes Mellitus				χ² = 11.00	0.004*
Yes	0 (0%)	13 (59.1%)	46 (63.0%)		
No	7 (100%)	9 (40.9%)	27 (37.0%)		
Chronic Kidney Disease				χ² = 0.39	0.821
Yes	0 (0%)	1 (4.5%)	7 (9.6%)		
No	7 (100%)	21 (95.5%)	66 (90.4%)		
Family History of IHD				Fisher’s Exact	1
Yes	0 (0%)	0 (0%)	1 (1.4%)		
No	7 (100%)	22 (100%)	72 (98.6%)		
Smoking Status				χ² = 0.95	0.621
Current smoker	0 (0%)	4 (18.2%)	8 (11.0%)		
Ex-smoker	0 (0%)	0 (0%)	5 (6.8%)		
Non-smoker	7 (100%)	18 (81.8%)	60 (82.2%)		
Anemia				χ² = 5.54	0.062
Yes	7 (100%)	12 (54.5%)	53 (72.6%)		
No	0 (0%)	10 (45.5%)	20 (27.4%)		

Mean serum creatinine was higher in the anemic group (1.26 ± 0.41 mg/dL) compared to non-anemic patients (0.99 ± 0.32 mg/dL; p = 0.002), while mean eGFR was markedly lower among anemic individuals (69.2 ± 17.5 vs. 87.4 ± 14.6 mL/min/1.73m²; p < 0.001). Reduced kidney function (eGFR <60 mL/min/1.73m²) was significantly more frequent in the anemic group (31.9% vs. 6.7%; p = 0.005) (Table 4).

**Table 4:** Comparison of Renal Function Between Anemic and Non-anemic HFpEF Patients (n = 102) * statistically significant

Variable	Anemia Yes (n = 72)	Anemia No (n = 30)	Test Statistic	p-value
Serum Creatinine (mg/dL), Mean ± SD	1.26 ± 0.41	0.99 ± 0.32	t = 3.27	0.002*
eGFR (mL/min/1.73m²), Mean ± SD	69.2 ± 17.5	87.4 ± 14.6	t = 4.89	<0.001*
eGFR <60 mL/min/1.73m², n (%)	23 (31.9%)	2 (6.7%)	χ² = 7.86	0.005*
Chronic Kidney Disease (clinical diagnosis), n (%)	5 (6.9%)	3 (10.0%)		0.69

## Discussion

This study evaluated the frequency of anemia and its clinical correlates among patients with HFpEF. The findings demonstrate that anemia is highly prevalent in this population, affecting 70.6% of patients, which is considerably higher than previously reported rates ranging between 27% and 58% in international cohorts. This elevated prevalence likely reflects the advanced age, high burden of comorbidities, and real-world tertiary care setting of the studied population, where patients often present with more severe and complex disease profiles. Anemia is commonly observed in patients with HF, regardless of whether they have preserved or reduced LVEF. However, previous studies have reported anemia prevalence rates among HFpEF patients ranging from 27% to 58% [19], which is notably lower than the rate observed in our study. In our cohort, over 70% of patients with HFpEF were anemic, based on World Health Organization (WHO) criteria. This higher prevalence may reflect real-world data specific to HFpEF patients. Notably, earlier reports have also suggested that anemia is more common in HFpEF than in HFrEF [20]. In our study, anemia in HFpEF was independently associated with several patient characteristics, including older age, female sex, impaired renal function, multiple comorbidities, frailty, and greater HF severity. These findings suggest that the high prevalence of anemia may reflect the distinctive clinical profile of patients with HFpEF. Although anemia in HF is associated with various contributing factors, its exact etiology remains largely unclear [21]. One recognized contributor is renal insufficiency; the coexistence of anemia, HF, and CKD is referred to as cardiorenal anemia syndrome. In this study, fewer than 8% of patients had a diagnosis of CKD. However, anemia was still associated with impaired renal function, as evidenced by significantly lower eGFR in anemic patients compared to non-anemic ones. Evaluation of the severity of diastolic dysfunction showed that the NT-proBNP levels and the E/e' ratios increased gradually with the increasing grades of dysfunctions as predicted, showing that filling pressures and the cardiac levels of stress are linked. However, anemia status itself never had a statistically significant relationship with categories of diastolic dysfunction, which implies that anemia may be better related to systemic and renal rather than direct cardiac structural impairment. This difference is also clinically significant because it suggests that anemia in HFpEF could be a comorbidity marker and not necessarily a sign of myocardial dysfunction. Clinically, the critical incidence of anemia in this group of patients highlights the importance of screening in HFpEF patients on a regular basis. Since hemoglobin testing is cheap, cost-effective, and easily accessible, early diagnosis and treatment of anemia, especially when accompanied by renal malfunction, could help improve the risk stratification and patient care optimization. The reversible causes that may lead to an improvement in functional capacity and quality of life in this population may be addressed by iron deficiency or renal impairment.

Limitations

This study has several limitations that should be considered. The use of non-probability sampling and exclusion of patients with alternative identifiable causes of anemia may have introduced selection bias and potentially inflated the observed prevalence. Being a single-center study conducted at a tertiary referral hospital, the cohort likely included older and more clinically complex patients, limiting generalizability to the broader HFpEF population. Detailed etiological characterization of anemia, including iron studies and red cell indices, was not routinely performed, restricting differentiation of anemia subtypes and clinical interpretation. The cross-sectional design prevents assessment of causal or temporal relationships between anemia, renal dysfunction, and HF severity.

## Conclusions

It is concluded that anemia is highly prevalent among patients with HFpEF, affecting more than two-thirds of the study population. Although anemia was not significantly associated with most clinical or echocardiographic indicators of HF severity, it demonstrated a strong relationship with impaired renal function, evidenced by higher serum creatinine levels and lower eGFR rates. These findings highlight anemia as a common and clinically relevant comorbidity in HFpEF rather than a direct marker of cardiac dysfunction.

## Appendices

Annexure-A

Proforma

Reg #: _______________                                        MR No: _________________

Name: _____________________________________________________________

Gender:      Male             Female       

Age: __________________ years                            BMI: ________________ Kg/m^2^

Height: _______________ meters                           Weight: _________________Kg

SBP: ________________ mmHg                             DBP:  _______________ mmHg

Trop I: ____________ ng/ml                                   LVEF: ________________________ %

NT pro BNP: ___________ pg/ml                           E/e’ ratio: _________

Co-morbidity:

ð      Hypertension

ð      Diabetes mellitus

ð      Family history of IHD

ð      Chronic kidney disease

Smoking status:        ___ Smoker        _____ Non-smoker       _____ Ex-smoker   

Hemoglobin level: ___________ gm/dl

Anemia:

ð Yes

ð No
